# Methods for Assessing Willingness to Try and Vegetable Consumption among Children in Indigenous Early Childcare Settings: The FRESH Study

**DOI:** 10.3390/nu14010058

**Published:** 2021-12-24

**Authors:** Marianna S. Wetherill, Mary B. Williams, Jessica Reese, Tori Taniguchi, Susan B. Sisson, Adrien D. Malek-Lasater, Charlotte V. Love, Valarie Blue Bird Jernigan

**Affiliations:** 1 Department of Health Promotion Sciences, Hudson College of Public Health, University of Oklahoma-Tulsa Schusterman Center, Tulsa, OK 74135, USA; 2 Department of Biostatistics and Epidemiology, Hudson College of Public Health, University of Oklahoma-Tulsa Schusterman Center, Tulsa, OK 74135, USA; 3 Department of Biostatistics and Epidemiology, Hudson College of Public Health, University of Oklahoma Health Sciences Center, Oklahoma City, OK 73117, USA; Jessica-Reese@ouhsc.edu; 4 Center for Indigenous Health Research and Policy, Oklahoma State University Center for Health Sciences, Tulsa, OK 74106, USA; tori.taniguchi@okstate.edu (T.T.); bluebird.jernigan@okstate.edu (V.B.B.J.); 5 Department of Nutritional Sciences, College of Allied Health, University of Oklahoma Health Sciences Center, Oklahoma City, OK 73117, USA; Susan-Sisson@ouhsc.edu; 6 Department of Teaching, Learning, and Curriculum, College of Education and Human Services, University of North Florida, Jacksonville, FL 32246, USA; a.malek@unf.edu; 7 School of Health Care Administration, Oklahoma State University Center for Health Sciences, Tulsa, OK 74107, USA; charlie.love@okstate.edu

**Keywords:** Native American, American Indian, dietary assessment, childhood obesity, community-based participatory research, childcare interventions, vegetables, food preferences

## Abstract

Food preferences begin in early childhood, and a child’s willingness to try (WTT) new vegetables is an important determinant of vegetable intake. Young children living in rural communities are at increased risk for food insecurity, which may limit exposure to and consumption opportunities for vegetables. This manuscript describes the validation of the Farfan-Ramirez WTT (FR-WTT) measure using baseline data from the FRESH study, a gardening intervention for Native American families with preschool-aged children in Osage Nation, Oklahoma. Individually weighed vegetable containers were prepared with six types of vegetables and ranch dip. Researchers presented children (*n* = 164; M = 4.3 years, SD = 0.8) with these vegetables preceding a snack- or lunch time and recorded the child’s FR-WTT for each vegetable using a 5-point scale, ranging from “did not remove food (0)” to “put food in mouth and swallowed (4)”. After the presentation period, contents were re-weighed to calculate vegetable consumption. Household parents/guardians completed the Child Food Neophobia Scale (CFNS) for their child. FR-WTT scores were positively correlated with consumption weights of all vegetables (r = 0.7613, *p* < 0.0001) and each vegetable individually (r = 0.2016–0.7664). The total FR-WTT score was inversely correlated with the CFNS score (r = 0.3268, *p* < 0.0001). Sensitivity analyses demonstrated similar relationships by BMI, food security, and age. In conclusion, the FR-WTT is a valid method for assessing young children’s vegetable eating behavior and intake.

## 1. Introduction

The preschool years are an influential time for the development of learned food preferences, which can ultimately shape lifelong eating habits [1,2,3]. Vegetables are a central component of healthy diets, and like many new or unfamiliar foods, many young children can often initially reject vegetables due to an increased sensitivity to their bitter taste [4]. The tendency to reject or be reluctant to try new foods, known as food neophobia [1], usually peaks between the ages of two and six years old [5], and is an important influence on a child’s food choices, preferences for and intake of vegetables [6], and overall quality of diet [7,8]. Interventions that repeatedly expose children to the same type of food may reduce food neophobia [9,10,11]. Thus, interventions that provide young children with multiple opportunities to taste a variety of vegetables may increase willingness to try new vegetables and overall intake of vegetables.

An estimated 59% of young children attend Early Care and Education (ECEs) programs [12], and these environments substantially contribute to a child’s dietary intake and exposure to new foods, such as vegetables [13,14,15]. Children from minority, rural, and lower-income households experience multiple nutritional disparities in healthy food access and consumption [16]. Thus, children who attend these ECEs may greatly benefit from healthy eating interventions [17]. However, researchers face unique methodological challenges for evaluating the impact of healthy eating interventions for young children in these settings. Most notably, children are unable to reliably self-report their own dietary intake [18,19], and errors in proxy reporting from parents or ECE caregivers can lead to biased estimates of intake [20]. When multiple caregivers participate in child feeding during the day, such as teachers, parents, and other child caretakers, these errors may be amplified. Weighed food records (plate waste) are the gold standard for assessing actual dietary intake of individuals [21]; however, this method is labor intensive and can be cost-prohibitive for large-scale intervention studies [22,23]. In addition, weighed plate waste may not be sensitive enough to detect subtle changes in a child’s willingness to try (WTT) a food, which includes willingness to taste, but not necessarily consume the food. Although multiple tools have been developed to measure school-aged children’s food neophobia [24,25], most are self-administered questionnaires [26], which cannot be administered to pre-school children and may be subject to proxy-reporting errors. Furthermore, these tools have also not been developed or validated to measure changes over time. Hence, a valid tool is needed to assess pre-school children’s WTT vegetables to better include the range of possible interactions with these foods.

Although researchers have developed various self-reported WTT tools for school-aged children [26,27], only one assesses young children’s WTT in ECE environments. Farfan-Ramirez and colleagues (2011) developed a five-point observational checklist to measure observed willingness to try (FR-WTT) specific fruits and vegetables among three- to five-year-olds, which could serve as an evaluation alternative to weighed plate waste, as well as to assess changes in neophobic food behaviors over time. This FR-WTT tool [28] served as an evaluation method for the “Nutrition Matters!” curriculum to assess preschoolers’ WTT for select fruits and vegetables (figs, raspberries, blanched snap peas, and roasted beets) [28]. Although the FR-WTT method has good face validity and many presumed benefits relative to more intensive dietary assessment methods, no validation studies have been conducted among preschool-aged children in ECE settings. The primary aim of this study was to validate the FR-WTT observational method for assessing two behavioral measures of acceptance for six different vegetables, willingness to taste and dietary consumption, among preschool-aged children in rural, Indigenous ECE settings.

## 2. Materials and Methods

### 2.1. FRESH Study Overview

This research was part of the Food Resource Equity and Sustainability for Health “FRESH” Study. We conducted this five-year community-based participatory research study collaboratively with the Osage Nation (ON), the Hudson College of Public Health at the University of Oklahoma Health Sciences Center (OUHSC), and the Center for Indigenous Health Research and Policy at the Oklahoma State University Center for Health Sciences. The purpose was to address nutrition-related obesity disparities among Native American families living in rural Oklahoma with young children attending one of nine ON tribally operated ECEs, including four ON Head Start programs, four ON Wah-Zha-Zhi Early Learning Academies (WELAs), and one ON Language Immersion School (Daposka Ahnkodapi). The University of Oklahoma Health Sciences Center Institutional Review Board (IRB) served as the IRB of record for the study, as per the request of the Osage Nation leadership, and approved all data collection procedures involved in this study. These analyses used the baseline data from the FRESH study.

### 2.2. Recruitment and Study Population

We implemented multiple strategies to recruit families with children enrolled at one of the nine ON ECE programs from August 2017 through to January 2018. Research staff recruited families at parent orientations, back-to-school nights, and at school drop-offs and pick-ups. Working with ECE leadership, we contacted remaining eligible adults via telephone to inform them about the study and invite them to participate. We distributed promotional study materials, such as a letter signed by the Osage Nation Chief, in children’s backpacks, through the mail and via email, and we posted the materials on school bulletin boards.

Households with one or more family members identifying as AI and having at least one child, aged three to six years, attending one of the nine participating ON ECE sites were eligible to enroll in the study. Following parental consent, we enrolled 176 children and at least one household caregiver (parent or guardian). We collected child dietary assessment measures, including weighed food consumption and FR-WTT observations, between December 2017 and February 2018 on 164 of the 176 (93%) enrolled children. We collected body composition measures between November 2017 and February 2018 on 159 children (90%). Household caregivers received compensation for their time for completing questionnaires regarding themselves and their children.

### 2.3. Demographics, Food Security and Biometric Measures

Upon enrollment, household caregivers completed questionnaires about their children and themselves. Questionnaire data used for analyses reported in this manuscript included demographics, such as the child’s age, sex, race/ethnicity, the highest education of the caregiver, caregiver’s employment, annual household income, as well as the United States Department of Agriculture (USDA) 18-item Household Food Security Survey module [29]. In addition, we followed a standard protocol to measure the child’s height and weight without shoes using the Hopkins Road Rod Portable Stadiometer (#680214) and Health o meter^®^ 349KLX medical weight scale, respectively.

### 2.4. Selection of Test Vegetables

The FRESH study selected six target vegetables (carrots, spinach, tomatoes, lima beans, bell peppers, and butternut squash) as the focus vegetables of a future ECE gardening curriculum intervention. Vegetables were selected based on varying degree of novelty, regional availability, cultural preferences, and cost using participatory methods with tribal partners described elsewhere [30]. Menus for the nine ECE programs were prepared by two central planning groups, one for the Head Start programs and one for the WELA and Language Immersion schools. The relative novelty of each vegetable was determined by examining a six-week cycle menu for both central planning groups and one-week, on-site observations of foods served at lunch for each of the nine ECE schools prior to the intervention. Butternut squash, raw tomatoes, and lima beans were not documented on menus or observations at any site. It is worth noting that lima beans can be included in mixed vegetable blends, but no vegetable blends observed on-site included lima beans. Raw spinach was not documented on any menu but observed as part of a lettuce–spinach salad blend at one site during observation. Bell pepper was documented on only the WELA menu, but not observed at any site. Raw carrots were documented on menus and observations at all sites.

### 2.5. Weighed Plate Waste

We measured target vegetable consumption by weighed plate waste as the gold standard for objectively measuring food intake. Research staff were trained by the study’s research dietitian using a standardized written protocol. Trained university research staff numbered, weighed, and prepared vegetable containers with standardized pieces of the six target vegetables. Vegetables were portioned so that each vegetable variety visually occupied equal space in the container. Containers included three raw grape tomatoes (M = 23.2 g), three raw baby carrots (M = 29.9 g), five fresh spinach leaves (M = 5.1 g), five cubes of roasted butternut squash (M = 29.1 g), five cooked lima beans (M = 15.0 g), and three slices of raw green bell pepper (M = 22.3 g) along with a 2 oz. pre-packaged container of ranch dip. Trained research staff weighed each vegetable container prior to and after the addition of each vegetable and the ranch dip container. We used these serial weights to calculate an objectively measured pre-presentation weight for each vegetable before they were served to the child. We used digital scales (Tanita model KW-002) to obtain all the weights.

We recorded container numbers for each child as they were served to children in their classrooms. Containers were provided immediately preceding a routine morning snack, lunch, or an afternoon snack period, and all children had previously received at least one meal or snack by the school earlier in the school day. The meals provided by the program were in compliance with the United States Department of Agriculture Child and Adult Care Food Program and were all similar in nature. Breakfast consists of three food components and included milk, a grain or meat/meat alternate (i.e., eggs), and a fruit or vegetable. Lunch consists of five components and includes milk, grain, meat/meat alternate, fruit and vegetable (or double vegetable). After the vegetable presentation, trained researchers replaced any vegetable particles not consumed back into the vegetable container, such as vegetables that had dropped on the floor or that were on the table or in the child’s chair. Next, trained research staff weighed each vegetable container with all remaining contents, then re-weighed after removing each vegetable and unconsumed dip, starting with the unconsumed dip. We used these post-presentation serial weights to measure the post-presentation weights for each vegetable. We estimated the weight of each child’s vegetable consumption as the weight difference between pre-presentation and post-presentation weights for each vegetable type (e.g., Consumption weight_tomatoes_ = pre-presentation weight_tomatoes_−post-presentation weight_tomatoes_) [31]. We estimated the total consumption weight for each child by summing the consumption weight of all six target vegetables.

### 2.6. Direct Observation of Willingness-to-Try Measures

All research staff conducting WTT observations had previously completed laboratory training and passed field observation testing for the Ball plate waste observation method [31], which required simultaneous observation of four preschool children by each researcher to visually estimate consumption of individual items from a mixed plate of food. For this study, these same research staff were oriented to the FR-WTT method by the research dietitian and trained using a written standardized protocol.

At the time that the containers were presented, the trained observers rated each child’s interaction with each vegetable in the vegetable container using the FR-WTT checklist [28]. The FR-WTT rating options are: (0) Did not remove vegetable from box, (1) removed food, but did not bring to nose/mouth, (2) removed food and brought to nose/mouth, but did not put food in mouth, (3) put food in mouth, but did not swallow food (including taking a bite and spitting it out or licking an item), (4) put food in mouth and swallowed [28]. The maximum ratio of observer to children was 1:4, which is consistent with ratios used in other direct observation methods of dietary intake in young children [31]. We calculated the total FR-WTT by adding the observer ratings (0–4) for each of the six vegetables, for a total possible score of 0–24 for all six vegetables.

### 2.7. Child Food Neophobia

Household caregivers completed the six-item Child Food Neophobia Scale (CFNS) for their children, which was adapted by Cooke and colleagues [32] from the original CFNS developed by Pliner [33]. A higher CNFS score has been associated with lower consumption of fruits and vegetables [6]. The CFNS asks caregivers to rate six of their child’s behaviors when presented with novel foods on a 4-point Likert scale. For example, the questionnaire asks household caregivers: “My child does not trust new foods”. The response options are: (1) Strongly disagree, (2) somewhat disagree, (3) somewhat agree, or (4) strongly agree with the statement. We calculated the total CFNS score by summing the individual responses (1–4) for all six questions included in the survey. The total scores ranged from 6 to 24, with higher scores indicating higher food neophobia.

### 2.8. Validation of FR-WTT Observational Method

We assessed the criterion validity of the FR-WTT observational scale using a combination of approaches. The first approach assessed whether children observed to eat the vegetable(s) had a consumption weight higher than those who were not observed to eat the vegetable(s). Specifically, children observed as not touching any of the vegetables (FR-WTT = 0), touching but not putting any vegetables in their mouth (FR-WTT = 1 or 2), or putting a vegetable in their mouth and spitting it out (FR-WTT = 3) would have a negligible measured weighed vegetable consumption. In contrast, children observed putting a vegetable in their mouth and swallowing (FR-WTT = 4) would have weighed vegetable consumption above zero. Therefore, to determine if the mean consumption weights differed significantly among children, we observed consuming each vegetable compared to children who did not; we re-categorized FR-WTT into two categories—consuming the vegetables (WTT = 4) and not consuming the vegetables (WTT = 0, 1, 2, or 3). We hypothesized consumption weights would be significantly higher for children with a FR-WTT of 4 compared to those with a FR-WTT of 0–3.

Secondly, since the CFNS is designed to identify children reluctant to try new foods and most young children infrequently eat a variety of vegetables in the US [34], we assessed convergent validity of the FR-WTT with the CFNS using correlations between the FR-WTT and the CFNS. Based on our literature review [1,6], we hypothesized children with higher food neophobia would be less likely to interact with and try the vegetables. Therefore, we expected to observe an inverse relationship between these two constructs.

### 2.9. Statistical Analyses

We used Statistical Analysis Software package (version 9.4, SAS Institute, Inc., Cary, NC, USA, 2016) to conduct statistical analyses. We estimated Body Mass Index (BMI) percentiles based on growth charts from the Centers for Disease Control and Prevention [35]. We defined child obesity as above the 95th percentile and overweight was defined as above the 85th and below the 95th percentile [35]. To describe continuous variables, we calculated means and standard deviations, and calculated percentages for categorical variables.

We used single-family analysis of variance (ANOVA) to determine if the mean vegetable weight consumption differed between children who did (FR-WTT = 4) and did not consume the vegetables (FR-WTT = 0–3). We assessed the criterion validity of the FR-WTT scale by assessing the correlation between the FR-WTT scores and the CFNS scores. Prior to assessing this convergent validity, we assessed the correlation between CFNS and weighed plate waste to confirm an inverse relationship of child neophobia and food consumption, as reported in previous literature [7,8]. Additionally, we examined the internal consistency of the FR-WTT scale using a split sample. We split vegetables into two similar groups with one red/orange vegetable (tomatoes or carrots), one green vegetable (peppers or spinach), and one cooked vegetable (squash or beans); then, we summed the WTT scores for each group. Next, we examined the correlation between the group WTT scores as a measure of internal consistency. Since the FR-WTT scores did not meet the assumptions for the Pearson correlation, we used Spearman correlations for all correlation analyses.

Finally, to examine the robustness of these analyses, we performed four sets of sensitivity analyses. First, to adjust for measurement error of consumption weights, we adjusted all negative vegetable consumption weights to zero and re-assessed the criterion validity of the FR-WTT with these adjusted consumption weights. Next, we performed three sets of analyses stratified by BMI, age, and food security groups to assess whether relationships were similar in these subgroups. We used single-family ANOVA to compare FR-WTT with consumption weights and CFNS in these stratified analyses.

## 3. Results

### 3.1. Participant Demographics, Food Security and Biometric Measures

A total of 164 children and their household caregivers enrolled in the study and completed the questionnaire. The mean age of the children included in our study was 4.7 years (*SD* = 0.8), 56% were female, 71% were Native American, 18% were overweight and 18% were obese. Among the adults caring for the children in our study, 44% had a high school education, 72% were employed, and 29% had a household income of USD 20,000 or less. In addition, 39% of children were living in households with low or very low food security and 17% were living with low or very low child food security (Table 1).

### 3.2. Description of Weighted Plate Waste, FR-WTT and CFNS

**Figure 1:**
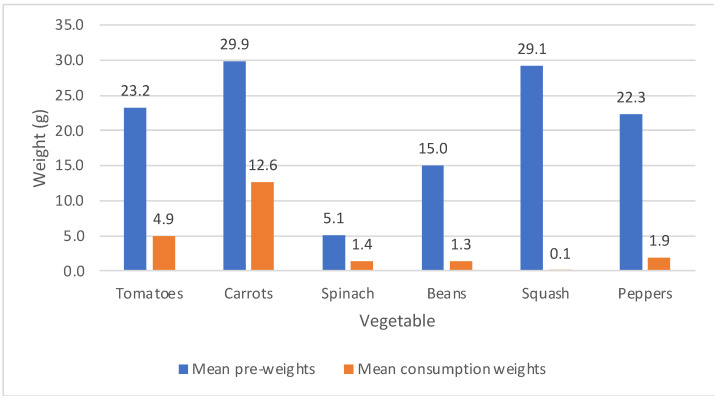
Mean pre-consumption weights and mean consumption weights for each vegetable.

For this study population, the total mean vegetable consumption was 22.2 g (*SD* = 21.8 g). On average, children consumed over 40% of the presented carrots (*M* = 12.6 g, *SD* = 12.8 g) over 20% of the presented spinach (M = 1.3 g, *SD* = 2.9 g) and tomatoes (M = 4.9 g, *SD* = 7.9 g), less than 10% of the presented beans (M = 1.9 g, *SD* = 4.5 g) and peppers (M = 1.4 g, *SD* = 1.9 g), with minimal consumption of the presented squash (Figure 1).

**Figure 2:**
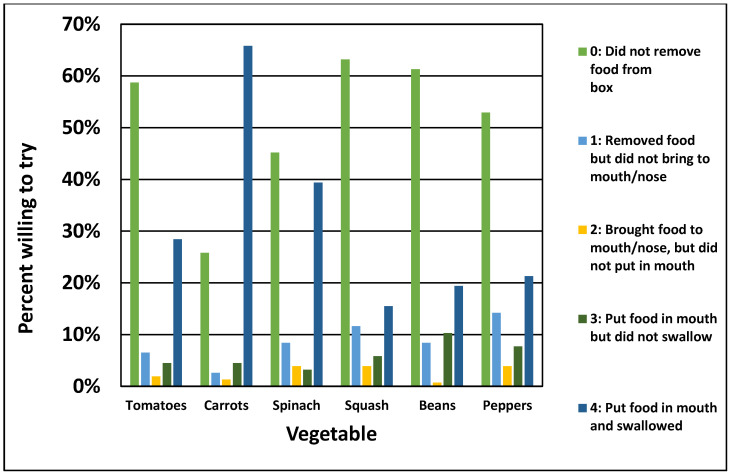
Percent of preschool children observed willing to try six vegetables according to the Farfan-Ramirez willingness to try (FR-WTT) scale.

The mean total FR-WTT score was 9.3 (*SD* = 7.3, range = 0–24). We observed only 5% of children placing all six vegetables into their mouths and swallowing and a little over one-quarter of children (30%) did not touch any of the vegetables. Figure 2 displays the distribution of children’s willingness-to-try behaviors according to the FR-WTT for each vegetable. More children were willing to try carrots (70%) and spinach (42%) by at least putting these vegetables in their mouths (FR-WTT scores of 3 or 4). Children were least willing to try squash and beans with 64% and 62% not removing these vegetables from the vegetable snack box, respectively.

The mean CFNS score reported by household caregivers was 14.0 (*SD* = 5.0, median = 14.0, range: 6–24) among the 146 children with CFNS scores. Seventeen children (12%) were rated at the lowest possible score on the CFNS scale (CFNS = 6) by their household caregiver (i.e., parents indicated that their children were willing to try new foods), 43% were rated with low neophobia (CFNS = 7–14) and 45% were rated with high neophobia (CFNS = 15–24) by household caregivers (i.e., parents indicated that their children were not willing to try new foods).

We did not find any differences in measures of interest (average vegetable weighed plate waste, total FR-WTT score, and total CFNS score) according to household caregiver race/ethnicity, child race/ethnicity, obesity, or household demographics (Table S1). Additionally, there were no differences in total CFNS score by the child’s age. However, older children consumed more vegetables by weight (t(df) = 2.97(161)*, p* < 0.01) and had higher total FR-WTT scores (t(df) = 2.33(161)*, p* = 0.02).

### 3.3. Criterion and Convergent Validity of Willingness-to-Try Method

Table 2 presents how the mean consumption weights differed among children who were observed consuming each vegetable versus those observed not consuming vegetables. Mean consumption weights among children observed swallowing the vegetables ranged between 3 and 19 g, while mean weights for children not removing vegetables were negligible.

Furthermore, correlation results to assess convergent validity of the FR-WTT with the CFNS score supported moderate to weak inverse correlations (Table 3) for all vegetables except beans. Proxy-reported CFNS scores were moderately and inversely correlated with total FR-WTT scores and with FR-WTT scores for peppers and carrots. CFNS scores were weakly and inversely correlated with FR-WTT scores for tomatoes, squash, and spinach. Additionally, the results of the total CFNS score were inversely and moderately correlated with total vegetable consumption.

In addition, the internal consistency was high for the FR-WTT scale based on a split sample analysis. The FR-WTT scores for two groups of vegetables, which each included a red or orange vegetable, a green vegetable, and a cooked vegetable, were highly correlated with a correlation coefficient of 0.73 (*p* < 0.0001).

### 3.4. Sensitivity Analysis by BMI and Food Security

We repeated these analyses to assess the validity of the FR-WTT among subgroups of children, including children with BMI classified as overweight/obese and normal, children in households experiencing food insecurity and no food insecurity, and older and younger children. The results of these sensitivity analysis found comparable correlations between FR-WTT and measured vegetable consumption weights between groups (Tables S1–S3). Total correlations for FR-WTT and vegetable consumption and for FR-WTT and CFNS scales were slightly stronger for obese/overweight children, for children living in food-insecure households, and for older children.

## 4. Discussion

Early childhood is a critical time for the development of healthy eating preferences, which has led public health researchers and interventionists to focus on this developmental period for the narrowing of nutrition-related intergenerational health disparities among marginalized populations. However, evaluating these interventions presents unique challenges, since most validated dietary assessment tools require participants to accurately recall and quantify food consumption. The validation of early childhood dietary assessment measures is essential for the advancement of evidence-based strategies that promote the willingness to try and, ultimately, the consumption of vegetables, a learned food preference in young children.

The present study establishes the FR-WTT is a valid method to assess willingness-to-try behaviors and consumption of a variety of vegetables. We established criterion validity of the FR-WTT with significant and moderate to strong positive correlations with the gold standard of weighed consumption for five of the six vegetables presented, which supports the FR-WTT’s use in the evaluation of WTT behaviors targeted at nutrition interventions for children aged three to six years old. The FR-WTT also offers several advantages for weighed plate waste, including potential to capture discrete changes in willingness to try over time and lower research burden.

Further, our analyses support the convergent construct validity of the total FR-WTT score as an observational method for identifying young children with food neophobia. FR-WTT was negatively correlated with CFNS scores reported by the household caregiver. Although these correlations were not strong, there may be multiple reasons for these findings. First, CFNS measures the construct of general willingness to try new foods, while the FR-WTT measures the child’s willingness to try specific vegetables at one point in time. The weak yet significant inverse correlations between CFNS and vegetable intake observed in our study are consistent with correlations in a previous study [6].

Sensitivity analyses additionally demonstrated that FR-WTT similarly correlates with vegetable intake among children with different BMIs, different household food security statuses, and older and younger preschool children. Given the vegetable intake disparities documented in both childhood obesity [36,37,38] and child food insecurity [39,40,41,42], researchers should consider the FR-WTT as a valid measure for these special populations.

Our research experience implementing the FR-WTT also supports its benefit as a lower-cost alternative to weighed plate waste. Although this FR-WTT method requires trained observers to evaluate children’s eating behaviors, it does not require pre- and post-weighing of each food item. Weighed plate waste methods are resource intensive, requiring a substantial amount of staff time and expensive scales to ensure accuracy. In addition, the FR-WTT scale has the ability to identify children who would only touch or taste (but not eat) vegetables, which cannot be determined through weighed plate waste.

This study has some limitations. Although we did not measure hunger for each child, all the children were provided breakfast or a morning snack at all ON ECE schools, so the children were presumed to have a similar level of hunger when the target vegetables were presented just prior to their usual snack or lunch. We were unable to measure the inter-rater reliability or test–retest reliability of the FR-WTT scale in the field due to the additional burden on the ECE sites and staffing requirements. However, we found relatively high internal consistency using a split half design with two groups of similar vegetables. Another limitation was that consumption weights for some target vegetables for a limited number of children had negative values, which indicates a lack of precision in the weighing procedures, possibly due to field data collection, and can introduce additional sources of error. However, only 15% of the weighed plate waste records had any negative consumption weights with the majority (76%) under one gram. Additionally, we performed sensitivity analysis using adjusted weights by setting negative weights to zero and found comparable correlations between the FR-WTT and vegetable consumption weights. Since we collected these study data in a community-based setting, vegetable containers were transported to and from the feeding site in coolers. However, some temperature changes may have occurred to the vegetables in transit that could affect weights (e.g., water evaporation from or absorption by vegetables). Potential moisture absorption may explain the negative consumption weights observed for the cooked butternut squash and the observed weaker correlations between the FR-WTT and consumption weights for this vegetable. Finally, the school menu review confirmed that all but one or two target vegetables (carrots for all sites, bell pepper only for WELA sites) were novel within the children’s school food environment. We did not capture information regarding whether any of the target vegetables were familiar to the children in their home environments, which could have been informative when assessing the correlations between the FR-WTT and the CFNS, since neophobia is the avoidance or reluctance to eat unfamiliar foods. If some children with high CFNS were more familiar with one or more of the target vegetables, their FR-WTT score for that vegetable may not correlate as highly with their CFNS. Despite our inability to identify familiarity with target vegetables at home, CFNS scores were moderately or weakly inversely correlated with the FR-WTT scores, which is similar to correlations of willingness to try novel foods and child food neophobia scales reported in a review article [43].

Despite these limitations, this study establishes that the FR-WTT is a valid method to assess willingness-to-try behaviors and consumption of a variety of vegetables in pre-school children. The study also contributes to the significant lack of high-quality, validated data available for low-income racial/ethnic communities, specifically Native Americans, and is the first of its kind to be implemented in a reservation setting. Further research should determine the predictive validity of FR-WTT score on total daily vegetable intake and overall dietary quality, since this study only evaluated the FR-WTT in estimating vegetable intake during a single eating period. Inclusion of children’s prior exposure to selected novel vegetables and parental feeding practices, specifically regarding encouragement to try new foods and vegetables, would be prudent to include in future studies examining WTT and dietary intake. The current literature shows that repeated exposure to vegetables as snacks is effective in increasing total vegetable intake in children [44,45,46].

## 5. Conclusions

The FR-WTT observational scale is a valid method for assessing vegetable intake among pre-school children during a single eating period. This study supports its use in the evaluation of community-based interventions designed to assess young children’s willingness to try and, ultimately, consume more vegetables.

## Figures and Tables

**Table 1:** Descriptive summary of child, adult, and household characteristics (*n* = 164).

Characteristic	Number (%) ^a^
Child characteristics	
Age (M, SD)	4.7 (0.8)
Sex	
Male	75 (46)
Female	87 (56)
Race/ethnicity	
Native American	115 (71)
White/Caucasian only	37 (23)
Other	10 (6)
Body Mass Index Percentile	
Above 95% (Obese)	28 (18)
85–95% (Overweight)	28 (18)
Below 85% (Normal or underweight)	103 (65)
Child food neophobia score (M, SD)	14.0 (5.0)
Adult and household characteristics	
Caregiver highest education	
Some high school/high school degree/GED	70 (44)
Technical/Vocational/Associate’s degree	50 (32)
Bachelor’s/Post-graduate degree	38 (24)
Caregiver employment	
Employed ^b^	114 (72)
Not employed	14 (9)
Retired/student/homemaker	30 (19)
Annual household income	
USD 20,000 and under	45 (29)
USD 20,001–35,000	36 (23)
USD 35,001–50,000	32 (21)
Over USD 50,000	43 (28)
Household food security	
Very low food security	12 (8)
Low food security	47 (31)
Marginal food security	27 (18)
High food security	65 (43)
Child food security	
Low or very low child food security	25 (17)
High or marginal child food security	126 (83)

^a^ Percentages adjusted for missing values. ^b^ Includes employed full time, employed part time, seasonally/occasionally employed, self-employed.

**Table 2:** Mean consumption of vegetables during observations by Farfan-Ramirez willingness-to-try (FR-WTT) categories.

Vegetable FR-WTT	*n*	Mean Consumption in Grams ^1^ (SD)	*p* Value
Tomatoes FR-WTT = 4	33	7.6 (7.6)	**<0.001**
Tomatoes FR-WTT = 0, 1, 2, 3	131	0.4 (0.9)	
Carrots FR-WTT = 4	108	18.9 (11.4)	**<0.001**
Carrots FR-WTT = 0, 1, 2, 3	56	0.4 (1.0)	
Spinach FR-WTT = 4	63	2.7 (2.2)	**<0.001**
Spinach FR-WTT = 0, 1, 2, 3	101	0.6 (0.9)	
Beans FR-WTT = 4	31	4.9 (5.0)	**<0.001**
Beans FR-WTT = 0, 1, 2, 3	133	0.5 (1.1)	
Squash FR-WTT = 4	25	4.1 (7.7)	**<0.001**
Squash FR-WTT = 0, 1, 2, 3	139	−0.6 (1.1)	
Peppers FR-WTT = 4	33	7.6 (7.6)	**<0.001**
Peppers FR-WTT = 0, 1, 2, 3	131	0.5 (0.9)	

^1^ Consumption values are rounded to one gram. Bold denotes significant *p* value < 0.05.

**Table 3:** Evaluation of correlations ^a^ between Farfan-Ramirez willingness-to-try (FR-WTT) vegetables and the Child Food Neophobia Scale (CFNS).

Vegetable	Correlation ^a^ between FR-WTT and CFNS Scales	
	Coefficient	*p* Value
Tomatoes	−0.2786	**<0.01**
Carrots	−0.3004	**<0.01**
Spinach	−0.1762	**0.03**
Beans	−0.0986	0.24
Squash	−0.2454	**<0.01**
Peppers	−0.3259	**<0.001**
Total	−0.3268	**<0.001**

^a^ Spearman correlation. Bold denotes significant *p* value < 0.05.

## Data Availability

The de-identified data presented in this study are available on reasonable request from the corresponding authors and can only be released following permission from the senior author and Osage Nation. The data are not publicly available to protect privacy of tribal citizen human subject participants.

## Supplementary Materials

The following are available online at https://www.mdpi.com/article/10.3390/nu14010058/s1, Table S1a: Sensitivity analysis of mean consumption of vegetables during observations by Farfan-Ramirez willingness-to-try (FR-WTT) categories by child’s Body Mass Index (BMI), Table S1b: Sensitivity analysis of criterion and convergent validity stratified by child’s Body Mass Index (BMI). Correlations between Farfan-Ramirez willingness-to-try (FR-WTT) scale and the Child Food Neophobia Scale (CFNS), Table S2a: Sensitivity analysis of mean consumption of vegetables during observations by Farfan-Ramirez willingness-to-try (FR-WTT) categories by household food security, Table S2b: Sensitivity analysis of criterion and convergent validity stratified by household food security. Correlations between Farfan-Ramirez willingness-to-try (FR-WTT) scale and the Child Food Neophobia Scale (CFNS), Table S3a: Sensitivity analysis of mean consumption of vegetables during observations by Farfan-Ramirez willingness-to-try (FR-WTT) categories by child’s age, Table S3b: Sensitivity analysis of criterion and convergent validity stratified by child’s age. Correlations between Farfan-Ramirez willingness-to-try (FR-WTT) scale and the Child Food Neophobia Scale (CFNS).
