# Supplementary material for: Methods for Assessing Willingness to Try and Vegetable Consumption among Children in Indigenous Early Childcare Settings: The FRESH Study
Source: Nutrients. 2021 Dec 24;14(1):58. doi: 10.3390/nu14010058 (PMC8746319; doi:10.3390/nu14010058)
Supplement: Supplementary file 1 [file nutrients-14-00058-s001.zip › nutrients-1484512-SI/Supplemental Table 1 FRESH WTT Rev JR.pdf]

Supplemental Table S1a. Sensitivity analysis of mean consumption of vegetables during observations by Farfan-Ramirez willingness-to-try (FR-WTT) categories by child's Body Mass Index (BMI)

Vegetable FR-WTT	n	Mean Consumption in grams[1] (SD)	P-value
Among overweight or obese children (BMI ≥ 85%) (n=56)			
Tomatoes FR-WTT=4	15	16.4 (8.2)	**<0.001**
Tomatoes FR-WTT=0, 1, 2, 3	41	1.1 (1.4)	
Carrots FR-WTT=4	35	16.8 (12.4)	**<0.001**
Carrots FR-WTT=0, 1, 2, 3	21	0.6 (1.2)	
Spinach FR-WTT=4	25	2.2 (2.3)	**0.004**
Spinach FR-WTT=0, 1, 2, 3	31	0.7 (1.0)	
Beans FR-WTT=4	10	4.1 (6.1)	0.086
Beans FR-WTT=0, 1, 2, 3	46	0.4 (1.0)	
Squash FR-WTT=4	8	9.4 (11.3)	**0.042**
Squash FR-WTT=0, 1, 2, 3	48	-0.6 (1.3)	
Peppers FR-WTT=4	13	7.5 (7.0)	**0.003**
Peppers FR-WTT=0, 1, 2, 3	43	0.3 (1.0)	
Among normal weight children (BMI < 85%) (n=103)			
Tomatoes FR-WTT=4	29	15.7 (8.1)	**<0.001**
Tomatoes FR-WTT=0, 1, 2, 3	74	0.8 (1.2)	
Carrots FR-WTT=4	73	19.9 (10.9)	**<0.001**
Carrots FR-WTT=0, 1, 2, 3	30	0.2 (0.7)	
Spinach FR-WTT=4	38	3.1 (2.2)	**<0.001**
Spinach FR-WTT=0, 1, 2, 3	65	0.5 (0.8)	
Beans FR-WTT=4	21	5.3 (4.5)	**<0.001**
Beans FR-WTT=0, 1, 2, 3	82	0.6 (1.1)	
Squash FR-WTT=4	17	1.6 (3.4)	**0.014**
Squash FR-WTT=0, 1, 2, 3	86	-0.6 (1.0)	
Peppers FR-WTT=4	20	7.6 (8.2)	**0.001**
Peppers FR-WTT=0, 1, 2, 3	83	0.6 (0.9)	

[1]Consumption values are rounded to one gram.
Bold denotes statistically significant difference (p<0.05).

Supplemental Table S1b. Sensitivity analysis of criterion and convergent validity stratified by child's Body Mass Index (BMI). Correlations between Farfan-Ramirez willingness-to-try (FR-WTT) scale and the Child Food Neophobia Scale (CFNS).

Vegetable	Correlation between FR-WTT and CFNS scales	
	Coefficient	P-value
Among overweight or obese children (BMI ≥ 85%) (n=56)		
Tomatoes	-0.2369	0.07
Carrots	**-0.3366**	**0.01**
Spinach	**-0.3453**	**0.01**
Beans	-0.0929	0.49
Squash	-0.2480	0.06
Peppers	**-0.4128**	**<0.01**
Total	**-0.3948**	**<0.01**
Among normal weight children (BMI < 85%) (n=103)		
Tomatoes	**-0.2189**	**0.04**
Carrots	**-0.2069**	**<0.05**
Spinach	-0.0395	0.71
Beans	-0.1064	0.31
Squash	**-0.2449**	**0.02**
Peppers	**-0.3008**	**<0.01**
Total	**-0.2130**	**0.04**

Bold denotes statistically significant difference ($p<0.05$).
