# Supplementary material for: Methods for Assessing Willingness to Try and Vegetable Consumption among Children in Indigenous Early Childcare Settings: The FRESH Study
Source: Nutrients. 2021 Dec 24;14(1):58. doi: 10.3390/nu14010058 (PMC8746319; doi:10.3390/nu14010058)
Supplement: Supplementary file 1 [file nutrients-14-00058-s001.zip › nutrients-1484512-SI/Supplemental Table 2 FRESH WTT Rev JR.pdf]

Supplemental Table S2a. Sensitivity analysis of mean consumption of vegetables during observations by Farfan-Ramirez willingness-to-try (FR-WTT) categories by household food security.

Vegetable FR-WTT	n	Mean Consumption in grams[1] (SD)	P-value
Among children living in households reporting low or very low food security (n=59)			
Tomatoes FR-WTT=4	16	16.6 (7.6)	<0.001
Tomatoes FR-WTT=0, 1, 2, 3	43	0.7 (1.1)	
Carrots FR-WTT=4	40	17.9 (10.5)	<0.001
Carrots FR-WTT=0, 1, 2, 3	19	0.5 (1.1)	
Spinach FR-WTT=4	19	2.7 (2.0)	<0.001
Spinach FR-WTT=0, 1, 2, 3	40	0.4 (1.0)	
Beans FR-WTT=4	11	5.5 (6.0)	0.017
Beans FR-WTT=0, 1, 2, 3	48	0.4 (1.3)	
Squash FR-WTT=4	7	1.1 (2.4)	0.156
Squash FR-WTT=0, 1, 2, 3	52	-0.3 (1.2)	
Peppers FR-WTT=4	11	7.2 (5.8)	0.003
Peppers FR-WTT=0, 1, 2, 3	48	0.3 (1.0)	
Among children living in households reporting food high and marginal security (n=92)			
Tomatoes FR-WTT=4	23	16.1 (7.4)	<0.001
Tomatoes FR-WTT=0, 1, 2, 3	69	1.1 (1.3)	
Carrots FR-WTT=4	61	19.8 (12.0)	<0.001
Carrots FR-WTT=0, 1, 2, 3	31	0.3 (0.9)	
Spinach FR-WTT=4	39	2.7 (2.2)	<0.001
Spinach FR-WTT=0, 1, 2, 3	53	0.7 (0.9)	
Beans FR-WTT=4	15	4.7 (4.1)	0.002
Beans FR-WTT=0, 1, 2, 3	77	0.5 (1.0)	
Squash FR-WTT=4	14	4.8 (8.5)	0.030
Squash FR-WTT=0, 1, 2, 3	78	-0.8 (1.1)	
Peppers FR-WTT=4	21	8.0 (8.6)	0.001
Peppers FR-WTT=0, 1, 2, 3	71	0.5 (0.9)	

[1]Consumption values are rounded to one gram.

Supplemental Table S2b. Sensitivity analysis of criterion and convergent validity stratified by household food security. Correlations between Farfan-Ramirez willingness-to-try (FR-WTT) scale and the Child Food Neophobia Scale (CFNS).

Vegetable	Correlation between FR-WTT and CFNS scales	
	Coefficient	P-value
Among children living in households reporting low or very low food security (n=59)		
Tomatoes	-0.2369	0.07
Carrots	**-0.3366**	**0.01**
Spinach	**-0.3453**	**0.01**
Beans	-0.0929	0.49
Squash	-0.2480	0.06
Peppers	**-0.4128**	**<0.01**
Total	**-0.3948**	**<0.01**
Among children living in households reporting food high and marginal security (n=92)		
Tomatoes	**-0.3082**	**<0.01**
Carrots	**-0.2910**	**0.01**
Spinach	-0.0839	0.44
Beans	-0.1146	0.29
Squash	**-0.2481**	**0.02**
Peppers	**-0.2820**	**0.02**
Total	**-0.3028**	**<0.01**
