# Supplementary material for: Methods for Assessing Willingness to Try and Vegetable Consumption among Children in Indigenous Early Childcare Settings: The FRESH Study
Source: Nutrients. 2021 Dec 24;14(1):58. doi: 10.3390/nu14010058 (PMC8746319; doi:10.3390/nu14010058)
Supplement: Supplementary file 1 [file nutrients-14-00058-s001.zip › nutrients-1484512-SI/Supplemental Table 3 FRESH WTT Rev JR.pdf]

Supplemental Table S3a. Sensitivity analysis of mean consumption of vegetables during observations by Farfan-Ramirez willingness-to-try (FR-WTT) categories by child's age.

**Vegetable FR-WTT**	**n**	**Mean Consumption in grams[1] (SD)**	**P-value**
Among younger children (<4.7 years), (n=89)			
Tomatoes FR-WTT=4	25	16.2 (8.2)	<0.001
Tomatoes FR-WTT=0, 1, 2, 3	64	1.0 (1.3)	
Carrots FR-WTT=4	59	14.8 (11.3)	<0.001
Carrots FR-WTT=0, 1, 2, 3	30	0.4 (0.9)	
Spinach FR-WTT=4	31	2.3 (2.1)	<0.001
Spinach FR-WTT=0, 1, 2, 3	58	0.5 (0.9)	
Beans FR-WTT=4	13	5.5 (5.3)	0.006
Beans FR-WTT=0, 1, 2, 3	76	0.6 (1.1)	
Squash FR-WTT=4	10	6.1 (9.1)	0.046
Squash FR-WTT=0, 1, 2, 3	79	-0.5 (1.0)	
Peppers FR-WTT=4	17	6.5 (6.8)	0.002
Peppers FR-WTT=0, 1, 2, 3	72	0.4 (1.0)	
Among older children (≥4.7 years), (n=73)			
Tomatoes FR-WTT=4	19	15.6 (7.9)	<0.001
Tomatoes FR-WTT=0, 1, 2, 3	54	0.8 (1.3)	
Carrots FR-WTT=4	49	23.7 (9.7)	<0.001
Carrots FR-WTT=0, 1, 2, 3	24	0.4 (1.1)	
Spinach FR-WTT=4	32	3.1 (2.3)	<0.001
Spinach FR-WTT=0, 1, 2, 3	41	0.7 (0.9)	
Beans FR-WTT=4	18	4.6 (4.9)	0.002
Beans FR-WTT=0, 1, 2, 3	55	0.5 (1.0)	
Squash FR-WTT=4	15	2.8 (6.5)	0.060
Squash FR-WTT=0, 1, 2, 3	58	-0.7 (1.2)	
Peppers FR-WTT=4	16	8.8 (8.4)	0.001
Peppers FR-WTT=0, 1, 2, 3	57	0.5 (0.8)	

[1]Consumption values are rounded.

Supplemental Table S3b. Sensitivity analysis of criterion and convergent validity stratified by child's age. Correlations between Farfan-Ramirez willingness-to-try (FR-WTT) scale and the Child Food Neophobia Scale (CFNS).

Vegetable	Correlation between FR-WTT and CFNS scales	
	Coefficient	P-value
Among younger children (<4.7 years), (n=89)		
Tomatoes	**-0.2611**	**0.02**
Carrots	**-0.2673**	**0.02**
Spinach	-0.1951	0.08
Beans	0.0562	0.62
Squash	-0.2174	0.05
Peppers	**-0.2919**	**0.02**
Total	**-0.3179**	**<0.01**
Among older children (≥4.7 years), (n=73)		
Tomatoes	**-0.3107**	**0.01**
Carrots	**-0.3419**	**0.01**
Spinach	-0.1593	0.20
Beans	**-0.2604**	**0.03**
Squash	**-0.2759**	**0.02**
Peppers	**-0.3684**	**<0.01**
Total	**-0.3464**	**<0.01**
